# The Effects of Ramadan Fasting on Anterior Segment Parameters, Visual Acuity and Intraocular Pressures of the Eye

**DOI:** 10.2174/1874364101711010152

**Published:** 2017-06-30

**Authors:** Ozlem Barut Selver, Melis Palamar, Kevser Gerceker, Sait Egrilmez, Ayse Yagci

**Affiliations:** Department of Ophthalmology, Ege University, Izmir, Turkey.

**Keywords:** Anterior chamber parameters, Intraocular pressure, Ramadan fasting, Visual acuity

## Abstract

**Objective:**

It is aimed to determine whether fasting during Ramadan has any significant effect on anterior chamber parameters, visual acuity and intraocular pressures.

**Methods:**

31 fasting (Group 1) and 30 non-fasting healthy volunteers (Group 2) were enrolled. All cases underwent an ophthalmological examination and anterior segment parameter evaluation (central corneal thickness (CCT), anterior chamber depth (ACD), anterior chamber volume (ACV), anterior chamber angle (ACA), pupil size) with Pentacam before and after the breaking of the Ramadan fast in Group 1, before and after dinner in Group 2.

**Results:**

The mean age was 43.35 ± 13.20 in Group 1, 43.17 ± 12.90 in Group 2 (p= 0.955). No significant difference was detected in any of the parameters in both groups.

**Conclusion:**

There is a need for more detailed and associated studies to understand better about the influence of Ramadan fast on various ocular parameters.

## INTRODUCTION

Ramadan fasting consists of refraining from eating or drinking during daylight hours for one month of the year due to the Islamic calendar among the Muslims [1].

The pattern of eating and drinking dramatically changes during the Ramadan festival and this may lead to physiological changes and/or foster some pathological conditions [2]. Many articles have been published about the physiological and pathological effects of Ramadan fasting [2-7]. Dehydration and relatively increased fluid intake during the time between sunset and dawn are the most prominent issues that have been investigated on ocular changes in the literature [8-12].

In this study, we aimed to determine whether fasting during Ramadan has any significant effect on anterior chamber parameters, best corrected visual acuity (BCVA) and intraocular pressure (IOP).

## MATERIALS AND METHODS

Thirty-one fasting volunteers (Group 1) and 30 non-fasting volunteers (Group 2) were enrolled. None of the volunteers had any systemic or ocular diseases, any topical or systemic drug use, and any intraocular surgery history. The study was complied with the Declaration of Helsinki. All cases underwent detailed ophthalmological examination including BCVA evaluation by Snellen chart, IOP with Goldman applanation tonometer, keratometric parameters and anterior segment parameters (central corneal thickness (CCT), anterior chamber depth (ACD), anterior chamber volume (ACV), anterior chamber angle (ACA), pupil size) evaluation by Scheimpflug camera system (Pentacam, Oculus Optikgerate GmbH, Wetzlar, Germany). All measurements were performed before and after the breaking of the Ramadan fast in Group 1, and before and after dinner in Group 2. The parameters in pre- and post-prandial periods were compared. The Statistical Package for the Social Sciences version 11.5.0 (SPSS Inc, Chicago, IL, USA) was used for statistical analysis.

## RESULTS

The mean age was 43.35±13.20 (range, 20-70) in Group 1 and 43.17 ± 12.9 (range, 18-65) in Group 2 (*p*=0.955). Male to female ratio was 13/18 in Group 1 and 13/17 in Group 2 (*p*=0.910). The fasting duration of all Group 1 cases was 15 hours.

No significant difference between IOP, ACD, ACV and ACA measurements, VA, mean keratometric values and pupil size was detected before and after the breaking of Ramadan fast in Group 1. Although pre-prandial CCT of Group 1 was slightly less than post-prandial, the difference was not statistically significant. There was not any significant difference in Group 2. All measurement values and statistical data assessment results were given in (Tables **1** and **2**) in detail.

## DISCUSSION

There is a considerable modification of eating and drinking habits during the Ramadan fast [1]. As all nutrition changes, fasting may yield physiological or pathological alterations in human body. There are only a few studies, which evaluate the ocular changes in Ramadan fast and most of them focus on the effect of fluctuations in fluid intake. Besides, some of the other pathophysiological changes such as, serum glucose, lipid values and cortisol levels are the main research subjects.

IOP alterations during fasting are the leading research topic of ocular effects of Ramadan. Brucculeri *et al.* [8] showed that water drinking has a temporary IOP increase effect. Consistent with this data, Dadeya *et al.* [9] reported a statistically significant decrease in IOP among fasting participants. In addition to the decreased fluid intake, they also proposed that the lessening of serum lipids might result in the inhibition of prostaglandin secretion and this leads to lower IOP values. Kerimoglu *et al.* [10] showed a decrease in IOP after 12 hours of dehydration. However, Kayikcioglu and Guler [11and Assadi *et al.* [12] reported no significant difference in IOP during fasting and non-fasting periods. Similarly, we have reported no significant difference for IOP. It is being thought that, during the fasting period, sympathetic hyperactivity, elevated serum free fatty acids and cortisol may cause IOP increase. However, dehydration and inhibition of prostaglandin secretion may cause IOP decrease. As a result of these hypotheses, one can conclude that there is no significant IOP change overall [12, 13].

Besides changes in IOP, visual acuity, refraction and anterior segment variations are also investigated. Kerimoglu *et al.* [10] showed a decrease in tear secretion after 12 hours of dehydration without any additional differences on anterior segment parameters. Assadi *et al.* [12] reported that Ramadan fasting does not profoundly affect refractive error or visual acuity values in healthy individuals. Kerimoglu *et al.* [10] indicated that fluid loading at the pre-dawn meal might increase both IOP and tear secretion in the early morning period as well. They did not report any differences in the corneal and anterior chamber parameters.

Herein, although not statistically significant, a slight difference in CCT values of fasting volunteers before and after the breaking of fast was detected. However, no other differences in the remaining parameters in both fasting and non-fasting volunteers were found. Due to the timing of the Ramadan feast, the period of not eating and not drinking was about 15 hours in our study. This relatively long period might have led us to find a difference in CCT parameters, differing from earlier reports.

In conclusion, probable CCT and other anterior segment parameter alterations might take place during Ramadan feast. As the Islamic calendar is a lunar calendar and Ramadan appears at a different period in each year, when the period occurs at summer season, especially at warmer climate areas, the effect of the dehydration might be more distinct. There is need for more detailed and associated studies to understand better about the influence of Ramadan fast on various ocular parameters.

## Figures and Tables

**Table 1:** Pre- and post-breaking the fast parameters in Group 1.

	**Pre–Breaking the Fast**	**Post–Breaking the Fast**	***P* Value**
R - IOP (mm Hg)	13.39 ± 2.50	13.90 ± 2.50	0.096
L - IOP (mm Hg)	13.68 ± 3.20	14.13 ± 3.00	0.210
R - BCVA	0.97 ± 0.61	0.98 ± 0.60	0.712
L - BCVA	0.98 ± 0.65	0.98 ± 0.60	0.922
R - CCT (μm)	549.02 ± 42.62	554.14 ± 48.04	0.058
L - CCT (μm)	553.16 ± 41.57	559.21 ± 48.57	0.052
R - ACD (mm)	2.90 ± 0.50	2.89 ± 0.49	0.118
L - ACD (mm)	2.93 ± 0.54	2.93 ± 0.53	0.752
R - ACV (mm^3^)	148.10 ± 38.17	148.58 ± 37.82	0.727
L - ACV (mm^3^)	146.87 ± 37.09	149.10 ± 38.60	0.258
R - ACA (degrees)	34.25 ± 5.77	35.02 ± 5.37	0.099
L - ACA (degrees)	34.93 ± 6.93	35.74 ± 6.91	0.209
R - Kmean (D)	43.43 ± 2.01	43.34 ± 1.88	0.122
L - Kmean (D)	43.35 ± 1.90	43.38 ± 1.92	0.663
R - Pupil size (mm)	3.14 ± 0.60	3.14 ± 0.60	0.940
L - Pupil size (mm)	3.11 ± 0.66	3.08 ± 0.65	0.687

R= Right eye, L= Left eye, IOP= Intraocular pressure, BCVA= Best corrected visual acuity, CCT= Central corneal thickness, ACD = Anterior chamber depth, ACV= Anterior chamber volume, ACA= Anterior chamber angle, Kmean= Mean keratometry

**Table 2:** Pre- and post-prandial parameters in Group 2.

	**Pre-Prandial**	**Post-Prandial**	***P* Value**
R - IOP (mm Hg)	15.4 ± 2.50	14.7 ± 2.50	0.067
L - IOP (mm Hg)	15.37 ± 2.50	15.2 ± 2.70	0.531
R - BCVA	0.93 ± 0.15	0.93 ± 0.14	1.000
L - BCVA	0.95 ± 0.12	0.95 ± 0.10	0.573
R - CCT (μm)	545.18 ± 49.39	549.23 ± 50.00	0.110
L - CCT (μm)	556.24 ± 50.80	554.11 ± 47.73	0.470
R - ACD (mm)	2.84 ± 0.39	2.81 ± 0.41	0.377
L - ACD (mm)	2.86 ± 0.39	2.86 ± 0.40	0.766
R - ACV (mm^3^)	146.27 ± 37.88	148.20 ± 36.68	0.131
L - ACV (mm^3^)	146.33 ± 39.59	144.90 ± 39.38	0.225
R - ACA (degrees)	34.87 ± 6.29	35.05 ± 6.48	0.638
L - ACA (degrees)	35.09 ± 5.70	34.97 ± 6.44	0.846
R - Kmean (D)	44.44 ± 1.72	44.57 ± 1.74	0.257
L - Kmean (D)	44.43 ± 1.68	44.39 ± 1.63	0.446
R - Pupil size (mm)	3.38 ± 0.65	3.35 ± 0.69	0.550
L - Pupil size (mm)	3.46 ± 0.67	3.43 ± 0.65	0.557

R= Right eye, L= Left eye, IOP= Intraocular pressure, BCVA= Best corrected visual acuity, CCT= Central corneal thickness, ACD= Anterior chamber depth, ACV= Anterior chamber volume, ACA= Anterior chamber angle, Kmean= Mean keratometry
